# Bioactive Glass Nanoparticles: From Synthesis to Materials Design for Biomedical Applications

**DOI:** 10.3390/ma9040288

**Published:** 2016-04-14

**Authors:** Charlotte Vichery, Jean-Marie Nedelec

**Affiliations:** 1 SIGMA Clermont, Institut de Chimie de Clermont-Ferrand, Université Clermont Auvergne, BP 10448, Clermont-Ferrand F-63000, France; jean-marie.nedelec@sigma-clermont.fr; 2 CNRS, UMR 6296, ICCF, Aubiere F-63178, France

**Keywords:** bioactive glass, nanoparticles, sol-gel, biomedical applications

## Abstract

Thanks to their high biocompatibility and bioactivity, bioactive glasses are very promising materials for soft and hard tissue repair and engineering. Because bioactivity and specific surface area intrinsically linked, the last decade has seen a focus on the development of highly porous and/or nano-sized materials. This review emphasizes the synthesis of bioactive glass nanoparticles and materials design strategies. The first part comprehensively covers mainly soft chemistry processes, which aim to obtain dispersible and monodispersed nanoparticles. The second part discusses the use of bioactive glass nanoparticles for medical applications, highlighting the design of materials. Mesoporous nanoparticles for drug delivery, injectable systems and scaffolds consisting of bioactive glass nanoparticles dispersed in a polymer, implant coatings and particle dispersions will be presented.

## 1. Introduction

Since their discovery in the 1970s by Hench, bioactive glasses have been the subject of intensive investigations as biomaterials for bone tissue repair and replacement [1]. Their attractiveness lies in their ability to chemically bond with host tissues that are directly related to their atomic structure. When a bioactive glass is implanted, it dissolves gradually and the ions released promote the growth of a carbonated hydroxyapatite layer at its surface. This dissolution mechanism is enhanced by the low connectivity of the SiO_2_ network, thanks to the presence of network modifiers, like sodium and calcium, leading to the formation of non-bridging silicon-oxygens bonds [2]. Briefly, surface Na and Ca cations are first exchanged with H^+^ from the biological fluid, creating Si-OH bonds. If present initially, phosphate ions can also be released. Then, more Si-OH bonds are formed because of the hydrolysis of Si-O-Si bonds due to a pH increase and they re-polymerize, creating a glass surface depleted in Na and Ca cations. The migration of Ca^2+^ and PO_4_^3−^ ions to the surface follows, inducing the formation of an amorphous calcium phosphate layer, which then crystallizes into biomimetic hydroxyapatite thanks to the incorporation of hydroxide and carbonate ions from the biological fluid [3,4].

Because bioactivity is directly related to the glass dissolution rate, it is obvious that it will also be dependent on its morphology. The higher the specific surface area, *i.e.*, the contact surface between the material and the physiological fluid, the greater the glass bioactivity. Strategies to obtain a significant specific surface area imply an increase in the porosity and/or a decrease in the size of the materials synthesized. With a high surface-to-volume ratio, bioactive glass nanoparticles (20–500 nm) are thus of tremendous interest not only because they present a larger specific surface area, but also a higher surface energy compared to micrometric-sized particles [5,6]. In addition, their nanometric size allows material shaping versatility. The bioactive nanoparticles can be dispersed in a polymer scaffold, used to create a bioactive coating on implants, dispersed or even to be directly injected. It is worth noting that upon decreasing their size, the dispersion of nanoparticles becomes increasingly difficult and can thus pose a challenge. Finally, it is worth noting that their size also permits them to be internalized by different type of cells (macrophages, bone cells, cancer cells, *etc.*). If well controlled and oriented, this could be very interesting for drug delivery and cancer treatment, for example.

This review will first present the strategies for producing bioactive glass nanoparticles, focusing mainly on the sol-gel process. In a second part, their fields of applications for hard tissue (bones, teeth) regeneration and repair, and also for soft tissue regeneration, wound healing and drug delivery will be described. The focus will always be placed on the design of the bioactive materials, and on their structure/properties relationships.

## 2. Synthesis of Bioactive Glass Nanoparticles

### 2.1. From Melt-Derived Glass to Sol-Gel Chemistry

The original bioactive glass developed by Hench has a composition of 45% SiO_2_, 24.5% Na_2_O, 24.5% CaO and 6% P_2_O_5_, and has been prepared through a conventional high-temperature melting process, *i.e.*, through the melting of oxides mixed together at more than 1300 °C followed by a quenching step [7]. Though this process does not permit nanoparticles to be obtained, high-temperature syntheses do not always result in large-scale objects: flame spray synthesis has been used to produce bioactive glass nanoparticles [8]. Metal-organic precursors has been mixed and fed into a flame reactor and the nanoparticles collected on a filter above the flame. This temperature quenching allowed the formation of 20–80 nm amorphous particles.

In the 1990s, soft chemistry strategies and particularly the sol-gel process emerged, providing a more versatile method to design bioactive glass nanoparticles. In contrast to the previously described routes, sol-gel technology allows the synthesis of bioactive glasses of equivalent composition but at a much lower temperature. This process is based on hydrolysis and condensation of molecular precursors (alkoxydes or salts), which lead to the formation at room temperature and ambient pressure of an inorganic polymeric network. Solvent being trapped within the network explains the gel-like texture. In the case of pure silica, the hydrolysis and poly-condensation of tetraethyl orthosilicate (TEOS) induces the formation of primary colloidal nanoparticles (sol), which later agglomerate to form a 3D, highly connected network in acidic conditions or spherical secondary particles in basic conditions (also known as the Stöber process [9]). Because glasses produced in both acidic and basic media are made of coalesced nanoparticles, they present a lot of inter-particular interstices [10]. Hence the glasses synthesized through this process have a higher porosity compared to melt-derived ones. This conclusion has also been confirmed for SiO_2_-CaO-(Na_2_O)-P_2_O_5_ bioactive glasses (the role of Na_2_O being to reduce the glass fusion temperature, it has not been used for the sol-gel process) while comparing the specific surface area and the porosity of particles of similar sizes obtained by a melt-quenching route and a sol-gel route. Sol-gel-derived particles present a pore volume and a specific surface area two orders of magnitude higher than the melt-derived ones [11]. This makes them highly attractive in the biomaterial fields since, as said before, bioactivity is linked to the specific surface area of the materials as it has a direct impact on glass dissolution and apatite formation rates. In addition, numerous Si-OH bonds are still present once the glass is formed as the low elaboration temperature does not promote oxolation reactions, resulting in a lower connectivity of sol-gel glasses compared to melt-quenched ones. It is also worth saying that the sol-gel process is highly versatile as particles size and morphology can be tuned while playing on the initial water/alcohol ratio, the nature of the alcohol, the type of precursor, and the concentration and the nature of the catalyst (HNO_3_, citric acid, HCl, NH_4_OH) [12]. In brief, using sol-gel chemistry, bioactivity can thus be controlled not only by the composition, but also by the process in itself.

### 2.2. Looking into the Core of Sol-Gel Strategies

To produce bioactive glass sol-gel nanoparticles, research groups have modified the classical Stöber process in order to introduce Ca^2+^ and sometimes PO_4_^3−^ ions into the silica network. Of course, it is also possible through a milling step to obtain nanoparticles from a silica monolith (top-down approach), but the particles are neither homogeneous in size nor in shape [13]. Common precursors for calcium and phosphorous are respectively calcium nitrate and triethylphosphate (Et_3_PO_4_) or diammonium hydrogen phosphate ((NH_4_)_2_HPO_4_). From reviewing the literature, two trends emerge.

1. The first one involves the synthesis of silica nanoparticles followed by Ca^2+^ (and PO_4_^3−^) adsorption onto their surface [14,15];
2. The second one introduces calcium (and phosphate) ions along with the silica precursor prior to increase the pH in order to form the particles. In the latter strategy:
  (i) TEOS/Ca^2+^(/PO_4_^3−^) acidic mixture is sometimes added to an ammoniac solution [15,16,17,18,19];
  (ii) and, on the contrary, sometimes concentrated NH_4_OH is dropped inside the TEOS/Ca^2+^(/PO_4_^3−^) acidic mixture [20,21,22,23].

**Figure 1:**
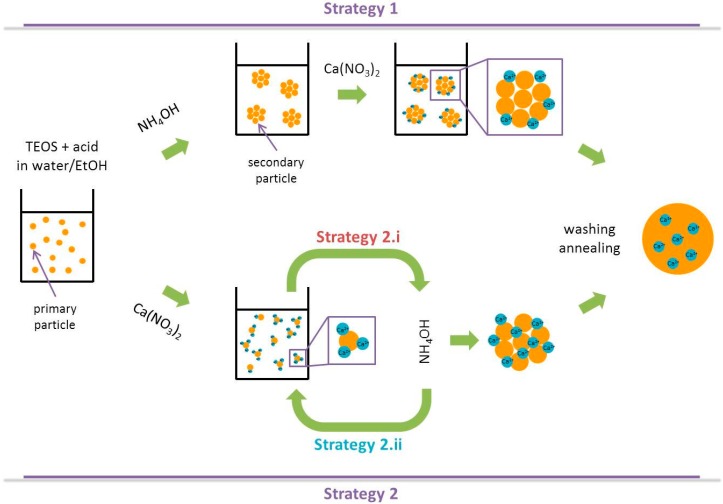
Schematic representation of the different strategies to synthesize binary bioactive glass nanoparticles (SiO_2_-CaO).

They are schematically represented in Figure 1.

**Figure 2:**
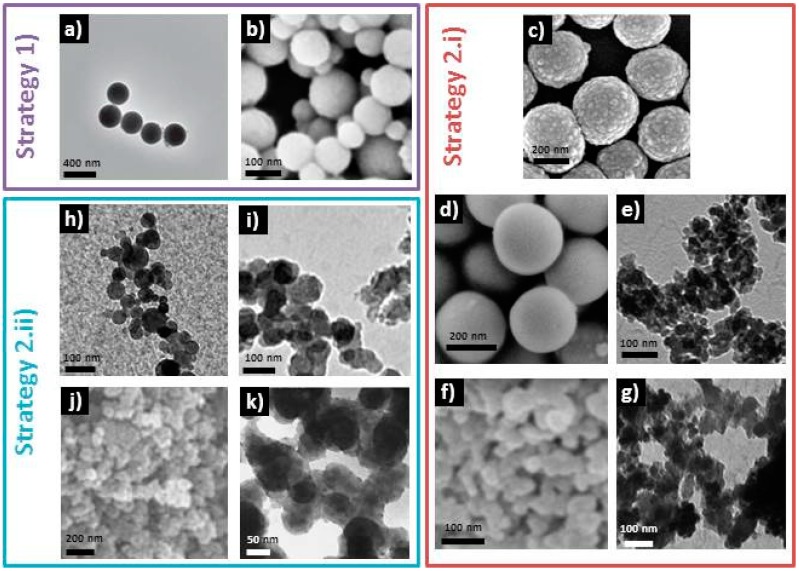
Selection of TEM or SEM images of bioactive glass nanoparticles synthesized by different groups. Adapted with permission from [14] (**a**); [16] (**c**); [17] (**g**), ©Wiley; [18] (**e**); [20] (**h**); [21] (**i**); [23] (**k**), ©Elsevier; [15] (**b**,**d**), ©Royal Society of Chemistry; [19] (**f**) and [22] (**j**), ©Springer.

The particles obtained in the syntheses referenced previously are shown in Figure 2. All these processes present common steps. For example, all differ from the original Stöber process because they add a hydrolysis step of TEOS in acidic media before accelerating the condensation and induce particle formation by the addition of concentrated NH_4_OH. Adding calcium ions after the formation of silica nanoparticles seems to be an efficient way to prevent them to interfere during the hydrolysis/condensation process and thus prevent the formation of spherical particles, as reported by Lukowiak *et al.* in comparative syntheses, where only the moment when Ca(NO_3_)_2_ salt is added has been varied [15]. However, it has also been shown that particles with controlled morphology can be obtained even if calcium salt is mixed with TEOS at the beginning of the synthesis (*cf.* Figure 2) [15,16]. One can see that it is extremely difficult to understand which are the parameters that have an actual impact on particles size and morphology, as all the protocols referenced here have different introduction precursors orders, different stabilization times between the addition of each one, different water/ethanol ratios and precursor concentrations, different types of acid (HNO_3_ or citric acid) and phosphorous precursors (Et_3_PO_4_ or (NH_4_)_2_HPO_4_), if there is one. Therefore, systematic studies are still needed in order to fully understand the role of each of the latter parameters.

Table 1 summarizes experimental conditions for the syntheses mentioned previously. For example, it has been shown that the Si:Ca ratio value was critical to obtain spherical particles, even when calcium is added after the formation of SiO_2_ particles [14]. This result is all the more important given that the Ca(NO_3_)_2_:TEOS optimal ratio was shown to be 0.57:0.43, which is far from the one usually used, and stoechiometrically identical to the desired particles composition, 0.3:0.7. Additionally, the calcium amount seems to impact the final size of the particles: as shown by Yun *et al.* in a synthesis involving hexadecyl trimethylammonium bromide (CTAB) as the template, increasing calcium nitrate quantity induced a decrease in diameter, from 160 to 30 nm [24].

Another common step to all the bioactive glass nanoparticle syntheses referenced above is a thermal annealing at 600–700 °C. This treatment is necessary to eliminate nitrate ions and to allow the diffusion of calcium ions inside the silica network. ^29^Si Magic Angle Spining (MAS)-NMR measurements realized onto bioactive glass before and after such thermal treatment have shown a decrease in silica network connectivity, hence proving that Ca^2+^ ions initially adsorbed onto the glass surface have been incorporated as network modifiers [10]. The necessity of such a high temperature heat treatment can lead to the agglomeration and coalescence of the as-synthesized particles, an effect that is all the more important as their size decreases, as their surface energy increases. One solution could be to use calcium methoxyethoxide as calcium source instead of calcium nitrate, as calcium would be directly involved in the inorganic polymerization process and thus inserted inside the silica network. Some groups successfully obtained bioactive glasses with calcium insertion in the network at low temperature [25,26]. However, to the knowledge of the authors, nanoparticles have not been yet produced using this alkoxyde precursor. It is important to mention that particle agglomeration is not only promoted because of the post-synthesis annealing step, but also by the decrease in particles’ surface charge during the synthesis. Pure silica particles have a zero charge pH value of about 2, meaning that for pH values lower than 2, their surface is positively charged (-OH_2_^+^ groups), and for pH values higher than 2, their surface is negatively charged (-O^−^ groups). The surface charge is characterized through the measure of zeta potential. Usually, it is considered that a zeta potential above |30| mV leads to the colloidal dispersion of particles in a medium. During bioactive glass synthesis, the pH value is quickly raised to ~11 in order to promote the formation of particles (Stöber process). Due to the high pH value, particles are electrostatically repulsed from each other and no agglomeration should occur. However, the addition of Ca^2+^ ions in the media and their adsorption at the particles surface decreases their surface charge and thus leads to particles agglomeration. In the literature, zeta potential values of −16.2 mV [27], −10 and −15 mV [19] at physiological pH have been reported after annealing. To minimize agglomeration, vigorous stirring is necessary, but most of syntheses still show a high degree of agglomeration (*cf.* Figure 2). However, carrying out the sol-gel process, and more particularly NH_4_OH addition, under ultrasonic treatment (in addition the mechanical stirring) has shown to lead to non-agglomerated bioactive glass nanoparticles [14]. Additionally, freeze-drying the particles prior to their annealing seems to give good chances for obtaining a colloidal suspension [16].

### 2.3. Of the Use of Additives and Surfactants

Still with the objective of shaping particles and preventing their agglomeration, surfactant and additives have been used by some groups. For example, Luz and Mano have shown that the addition of Polyethylene glycol (PEG )chains of different molecular weights at the end of the sol-gel process induced a change in particles morphology: smaller molecular chains PEG resulted in the formation of dense particles, whereas larger chains resulted in more agglomerated hollow particles [19]. Boltorn™ (Perstorp, Malmö, Sweden) polymer has also be used as a template by one group in an attempt to avoid particles agglomeration [28]. More successfully, a long chain amine (dodecylamine)-assisted sol-gel route has led to 200–300 nm spherical particles with a good dispersibility in water and ethanol solution [29]. CTAB is another templating agent that has been used to shape particles. It has been reported that increasing CTAB concentration from 1 to 6 mM changed the shape of the particles from spherical to rod-like [30]. Another study showed that increasing CTAB concentration from 3.3 to 5.9 mM leads to a decrease in the size of the particles, from 294 to 187 nm, and more importantly to a change in their structure, as for CTAB concentrations of 3.3 and 4.6 mM the particles were hollow, and for 5.9 mM they were dense [31]. One can note that CTAB concentration alone is not the only parameter which defines the morphology of obtained particles. In these two studies, even if the protocols were highly similar, precursor concentrations and the solvent were different. This again highlights the need of systematic studies to fully comprehend the role of each parameter on the particles morphology.

Synthetizing bioactive glass nanoparticles in micro-emulsions or nano-reactors is another route which has been explored in order to minimize their agglomeration. The principle is that a sol-gel-like reaction will take place in droplets formed by surfactants. Using poly(styrene-*b*-acrylic acid) and CTAB, Li *et al.* have obtained monodispersed 250 nm hollowed particles [32]. With a similar protocol, but using ethyl acetate along with CTAB, Liang *et al.* also obtained mesoporous well-defined particles, with a size ranging from 133 to 254 nm depending on the ammonia concentration used [33]. Finally, by a water-in-oil micro-emulsion route using Triton-100 as surfactant, *n*-hexanol as co-surfactant and cyclohexane as organic media, Lukowiak *et al.* synthesized monodispersed hollowed 90 nm nanoparticles which can be well dispersed in ethanol [15].

## 3. Materials Design for Biomedical Applications

The first part of this review focused on the preparation of bioactive glass nanoparticles through soft chemistry processes. But these particles will often not be used as-synthesized for biomedical applications. The second part will thus deal with the design of materials for biomedical applications, *i.e.*, the conception and elaboration of materials with specific structures, morphologies, tailored with the aim to use them for precise clinical purposes.

### 3.1. Nanoparticles for Drug Delivery and Ionic Therapy

Bioactive glass nanoparticles produced by sol-gel chemistry present high specific surface area and are biocompatible; they are thus good candidate to be carriers for drug delivery. Although bioactive sol-gel glasses have an inherent mesoporosity, it is possible, by combining sol-gel and surfactant supramolecular chemistries, to obtain ordered mesopore structures, presenting even a higher specific surface area [34]. When mixed inside the sol with an appropriate concentration, surfactants like CTAB, or pluronics F127^®^ or P123^®^ (BASF, Florham Park, NJ, USA) will self-organize into micelles. Along with the gelation process, the micelles will auto-assemble (evaporation-induced self-assembly), and after a thermal treatment, bioactive glasses with well-ordered mesopores will be obtained. Pore organization and sizes thus depend on both the surfactant used and its concentration [34,35], and due to its interaction with Ca^2+^ ions, to the composition of the bioactive glass [36]. To higlight the dependence of pore size, pores arrangement, particle morphology and thus the specific surface area of mesoporous bioactive glasses on the glass composition and on the surfactant nature and concentration, all these characteristics have been listed in Table 2 for a selection of studies. Adding 1–6 mM of CTAB to the sol-gel synthesis, spherical or elongated particles of sizes ranging from 30 to 300 nm were successfully synthesized [24,30,31]. It has been shown that the particles size was dependent on surfactant [30,31] and calcium [24] concentrations. With the addition of ethyl acetate along with CTAB, Liang *et al.* have obtained spherical particles with radial mesostructure, with a size ranging from 130 to 250 nm, depending on ammoniac concentration [33]. More interestingly, they showed that a solvothermal treatment induced the formation of pineal nanoparticles with a lamellar mesostructure, particles with an aspect ratio depending on the ammoniac concentration [33]. Hollow 250 nm particles with 20 nm pores inside the core and a vertical mesostructured 50 nm-thick shell have been synthesized using a dual soft template route using poly(styrene-*b*-acrylic acid) and CTAB [32]. This study distinguishes itself from the previously cited ones on the analysis of a cancer drug (doxorubicin) load and release. The particles present a remarkably high drug loading capacity (830 mg/g) and undergo a two-stage release linked to the particles peculiar morphology. It has been explained by a fast release of drug loaded in the mesoporous shell and a slower release due to the diffusion of drug molecules from the hollow core to the shell and then to the solution.

It is also interesting to note that drug loading and release kinetics can be controlled by bioactive glass calcium content [38]. Increasing the CaO quantity from 0% to 25% induced an increase in tetracycline (antibiotic) loading capacity from 105 to 174 mg/g and a decrease in drug release content from 95% to 25% after 120 h soaking in simulated body fluid. These results have been explained by the chelation of the drug to calcium species in the pore walls. The slow drug release of bioactive glass particles compared to pure silica ones is of high interest because it could prevent an undesirable burst effect. Loading drug while soaking bioactive glass particles in simulated body fluid could also be a way of slowing down drug release, thanks to the growth of a biological apatite layer onto their surface, as it has been shown on microspheres with bovine serum albumin [37]. Functionalizing the particles with amine groups, which have been shown to change particles surface charge from negative to positive [39], could also be a way to increase drug loading if the drugs involved are negatively charged.

If injected in the blood stream, the nanometric size of the drug carriers is of tremendous importance, as it determines their circulation half-life and cell uptake. Half-life is an important parameter, especially for particles not functionalized to target a specific marker, as it allows them to accumulate in the lesion before being cleared out of the body by the kidneys or the spleen. Particles specific accumulation into tumor tissues is called the Enhanced Permeability and Retention (EPR) effect, and lays on the presence of openings of 100–800 nm in tumor vasculature [40]. Smaller particles, diffuse in tumor tissue, can thus have some retention thanks to the high interstitial pressure inside the tumor, and possibly undergo cell uptake before being cleared by the mononuclear phagocyte system. However, particles should neither be too small, as under about 50 nm they pass through the vascular endothelium and are distributed in all over the body, non-specifically, and also because their dispersion will be more difficult, having a higher surface charge; neither will particles that are too big, as hydrodynamic stability will not be obtained in the blood stream. Hence, monodispersed particles with sizes between 50 and 200 nm should be used. It is important to say that bioactive glass nanoparticles can be internalized by human bone marrow and adipose-derived stem cells without significantly inhibiting their metabolic activities [14]. It thus shows that drug delivery could be achieved to help with bone tumor destruction and bone reconstruction.

Passive targeting will only be efficient for large tumors or lesion showing strong angiogenesis. For metastatic lesions or if drug delivery is undergone with another aim than destroying cancerous cells, active targeting through molecular recognition will be necessary. Composite nanoparticles with a stimuli-responsive polymer containing the drug could also be a possibility if the stimuli can be applied locally [41]. Another strategy could be a different injection system, with a direct implantation of the particles in the desired tissues. For example, Couto *et al.* have developed a really promising bioactive and biodegradable chitosan-based injectable system containing bioactive glass nanoparticles [42]. With a 50–100 nm sized nanoparticle quantity of 50%, the hydrogel has a gelation point of 36.8 °C, adequate for intracorporal injection. Also, the nanometric size of the particles allows the hydrogel to pass through small-gauge needles into bone defects. The bioactivity of this composite has been confirmed with the growth of a hydroxyapatite layer after three days in simulated body fluid. A thermo-responsive injectable system, with chitosan, bioactive glass nanoparticles and collagen has also been obtained by another group [43], and another one synthesized an injectable system in the form of 1.2 mm-diameter spheres, made of chitosan and bioactive glass nanoparticles crosslinked with genipin [44].

Because of the mechanism of bioactive glass dissolution and biological apatite growth, not only drugs but ions can be released. This process has some advantages like a local increase in pH due to the surface reactions involved, which was shown to have an efficient anti-bacterial effect against some oral bacteria [45]. However, more importantly, with the scope of ionic release and thanks to the versatility of sol-gel chemistry, glasses doped with metal ions are now produced with the aim of triggering specific biological responses. As extensively reported in a previous review, increased osteogenesis (Zn, Mg, Sr, Li and B ions), angiogenesis (Co and Cu ions), antibacterial (Cu and Ag ions) and anti-inflammatory (Zn and Sr ions) effects can be obtained [46]. Bioactive glass nanoparticles also have been successfully synthesized with Ag [21,22], or Zn [47] ions as dopants. El-Kady *et al.* performed a fine study of silver release rates for samples containing 1% to 10% mol of dopant [21]. They found out that the extraction of silver ions from the glasses followed a diffusion-controlled mechanism with release rates from 0.49 to 0.28 mg/L·h^−1^, respectively. The smaller quantity of Ag^+^ released for glasses containing higher amounts of dopant has been explained by a modification of the network connectivity, the replacement of Ca^2+^ by Ag^+^ reducing the number of non-bridging oxygen groups, thus decreasing glass dissolution rate. It is also worth noting that nanoparticles doped with rare earth ions presenting luminescent properties, which could thus be used to monitor intracellular processes, have been synthesized by some groups [15,29].

### 3.2. Polymer-Nanoparticles Composite Scaffolds

Bioactive glasses have been shown to be osteo-inductive, their dissolution products enhancing osteoblasts differentiation, upregulating the expression of genes playing a role in osteoblasts metabolism, proliferation and adhesion [48,49,50,51,52]. Bioactive glasses nanoparticles are thus highly attractive for bone tissue repair and regeneration. This is all the more attractive given that a recent study showed a better promotion of *in vitro* osteoblast-like cells and an earlier and increased expression of some osteogenic marker genes for submicron compared to micron-sized particles [5]. However, if nanoparticles can be directly injected in small bone defects, they cannot be used this way if the aim is to repair large bone defects, as the hydroxyapatite structure obtained will not be porous enough to permit the migration and proliferation of osteoblasts and mesenchymal cells and a good vascularization of the newly formed bone. Biomimetic structures are thus necessary for an optimal osteo-integration. In this scope, highly porous bioactive glass monoliths have been synthesized. Another way to obtain such macroporous structures is to disperse bioactive glass nanoparticles in a polymer matrix with an appropriate shape, in short, synthesizing macroporous composite scaffolds. The aim of adding nanoparticles into the polymeric structure is thus both to increase their mechanical properties that are intrinsically quite poor and provide a good bioactivity and osteo-induction while maintaining the polymer properties, such as flexibility.

**Figure 3:**
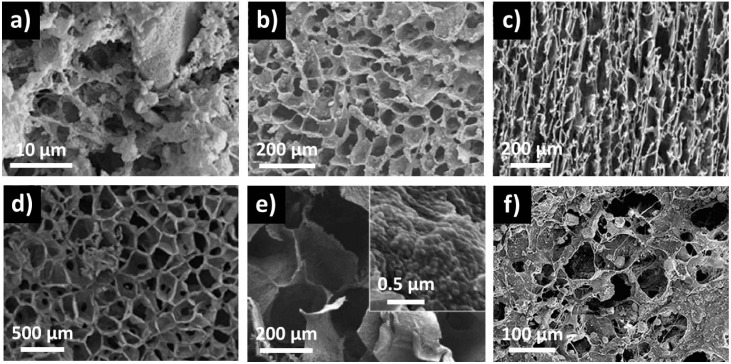
SEM micrographs of bioactive glass particles-polymer composites made by thermally-induced phase separation (TIPS), with poly(l-lactide), [20] (**a**) and [63] (**b**); poly(lactide-*co*-glycolide), [61] (**c**); chitosan-gelatin, [58] (**d**); gelatin, [59] (**e**) and collagen-phosphatidyserine, [62] (**f**) as polymer matrix. Figure adapted with permission from [20,58,61,62,63] ©Elsevier, [59] ©American chemical Society.

Different methods have been used to produce non-cytotoxic polymer-bioactive glass particles composites, but some like melt blending and thermal injection molding [53], twin-screw extrusion [54,55] or solvent casting [6], do not permit macroporous structures to be ontained. However, phase separation [56], lyophilization [57] and thermally-induced phase separation (TIPS), [20,58,59,60,61,62,63] (a variation of freeze-drying) processes have shown to allow the formation of interconnected pore structures with pore sizes from 10 to 300 µm. Considering the TIPS process, composite morphology depends mainly on the polymer used, as presented in Figure 3. In the TIPS process, bioactive particles are first mixed with a polymer and a decrease in temperature induced a separation into two phases. The solvent-rich phase is then removed by extrusion or sublimation, giving rise to open pores, while the composite-rich phase solidifies, creating the macroporous scaffold. The polymers used can be natural, like chitin [57]; chitosan-gelatin [58]; gelatin [59]; collagen-phosphatidylserine [62] or synthetic like poly(l-lactic acid) [20,56,63,64]; poly(lactide-*co*-glycolide) [60,61] or poly(d,l-lactide) [60]. All these studies have shown an increase in mechanical properties along with the addition of bioactive glass particles and their quantity inside the composite. However, the compressive strength and modulus measured are low compared to those of natural bones. The size of bioactive glass particles has been shown to have a strong impact on the mechanical properties of nanoparticle-gelatin composite scaffolds [59]. For example, for composites with a particles-polymer weight ratio of 50:50, adding 14 nm particles led to an increase in yield strength from 1.8 to 15.4 MPa. Increasing the size of the particles to 65, 106, 580 and 946 nm induced a decrease of yield strength to 10.9, 10.5, 2.1 and 0.8 MPa, respectively. It is worth noticing that, in this study, mechanical properties similar to cancellous bones have been obtained. The authors attributed these good mechanical properties to a homogeneous dispersion of the particles inside the polymer and to strong interactions between particles and gelatin, thanks to the presence of surface silanol groups, still present at the particles surface because of the absence of high temperature processing. Other studies have shown the impact of particles-polymer covalent bonding on the composites mechanical properties while functionalizing the nanoparticles with low molecular-weight poly(l-lactide) through diisocyanate coupling [64] or 3-aminopropyl trimethoxysilane [56]. As previously mentioned, not only particle-polymer interactions matters but also particles dispersion into the matrix. Liu *et al.* [64] showed that while increasing the bioactive particles loading in poly(l-lactide), the composite tensile strength decreased due to a severe aggregation of the particles.

Apart from enhancing mechanical compressive strength, the use of bioactive glass nanoparticles to obtain bioactive composite also induces improved bioactive properties. This can be explained by a morphology mimicking the nanoscale features of natural bones, which has been proven to increase osteoblast adhesion and proliferation [65]. Of course, this argument is valid only if nanoparticles are present on the polymer surface, with a direct contact with body fluids, thus emphasizing again the importance of homogeneous particles dispersion inside the polymer, related to the synthesis of non-agglomerated objects. A second reason is the enhanced bioactivity of particles with nanometric sizes compared to micrometric ones, which will be progressively exposed to body fluids while the polymer degrades. Ideally, a congruent dissolution of both polymer and bioactive glass nanoparticles should occur in order to obtain a biological hydroxyapatite scaffold with a similar morphology to the one of the composite. Also an equivalent degradation of particles and polymer should maintain the mechanical properties as new bone grows. The use of bioactive glasses is again highly interesting as their dissolution products increase locally body fluids pH. It can thus counter the decrease in pH induced by synthetic polymers hydrolysis [60,61]. It is also worth mentioning that the bioactive glass nanoparticles used to form the composites can be loaded with drugs or doped with metal ions to add the benefits of local antibacterial, anti-tumoral or pro-angiogenesis drugs/ions release to hard tissue regeneration and repair.

The biomedical applications of such bioactive glass nanoparticles-polymer composites are mainly for hard tissue engineering, in orthopedics or dentistry (periodontal bone regeneration). But applications for soft tissue repair have also been proposed [66]. One example, which is well described in the latter review, is the development of composites for heart patches which could provide mechanical support to the heart and help tissue repair after damages linked to the blockage of the coronary arteries irrigating the heart. With this scope, Chen *et al.* have created bioactive glass nanoparticle—poly(glycerol sebacate) composites with good mechanical properties, flexibility and biocompatibility [67]. Composites also have shown good results for wound dressing. For example Rai *et al.* have synthesized bioactive glass nanoparticles-poly(3-hydroxyoctanoate) composite films which have shown to decrease blood clotting time [68]. In addition to hemostatic properties, composite films can help with soft tissue repair, as demonstrated by Day *et al.* with polyglycolic acid meshes coated with bioactive glass micron-sized particles subcutaneously implanted in rats [69]. After 42 days, they presented complete tissue infiltration and neovascularization. Also, bioactive glass nanoparticle–gelatin composite conduits have be shown to be good candidates to help peripheral nerve regeneration [70]. Considering the poor regenerative capabilities of adult lung tissue, bioactive glass particle-polymer composites have been considered for lung tissue engineering, as it could provide a support for lung cells proliferation and adhesion. A poly(d,l-lactic acid) porous foam loaded with 5% bioactive glass micron-sized particles, for example, has been shown to be a promising candidate [71].

### 3.3. Implants Coating

Even though bioactive scaffolds with good mechanical properties have been produced, their use for load-bearing application is still a challenge. In order to protect against corrosion and solve the problem of bonding with tissues, the idea of coating metallic implants with bioactive glass has emerged. The electrophoretic deposition (EPD) technique involves the use of bioactive glass nanoparticles. This technique has the advantage of creating conformal coatings of single- or multi-phase particles onto a conductive substrate. Using EPD, bioactive glass microparticles dispersed in water have been deposited, the most uniform coatings resulting from electrodeposition in neutral solutions [72]. Another group has shown the feasibility of co-depositing carbon nanotubes and bioactive glass particles in order to create nanostructured composite coatings [73]. Additionally, adhesive several micrometer thick composite coatings made of bioactive glass particles dispersed in chitosan or alginate have been produced by EPD [74]. Very recently, Rego *et al.* realized bioactive coatings with an architecture similar to nacre using bioactive glass nanoparticles, chitosan and hyaluronic acid modified with catechol groups through layer-by-layer deposition [75]. These coatings have shown excellent adhesive properties and good bioactivity, and could thus be used as coating for implants in dentistry or orthopedics.

In addition to metallic implants, coatings using bioactive glass nanoparticles have been performed onto ceramic scaffolds. Not only an increased bioactivity, but also enhanced mechanical properties have been obtained. For example, a 14-fold increase in compressive strength and a 3-fold increase in compressive modulus have been achieved by coating hydroxyapatite/β-tricalcium phosphate scaffolds with a composite of 40 nm sized bioactive glass particles in polycaprolactone (30 wt%) [76]. A 6.5-fold increase in compressive strength (from 0.22 to 1.49 MPa) has also been achieved by coating a piece of cleaned cancellous bone extracted from an adult bovine femur with bioactive glass nanoparticles dispersed in polyvinyl alcohol [77]. In both cases, the macro-porous structure of the scaffold has been unaltered by the coating process. 

### 3.4. Dispersed Nanoparticles for Dentistry and Wound Healing

Bioactive glass particles have also found applications as dispersion into a body fluid like blood or saliva, or in paste like Vaseline. Exploring new fields of applications, it has been found that bioactive glass particles present good hemostatic properties, *i.e.*, while mixed with blood, they decrease its clotting time and increase its coagulation rate. This could be explained by the release of Ca^2+^ ions, which play a role in fibrin polymerization and clot stabilization [78]. Quickly mixed with blood, they could thus prevent large blood loss. Their positive impact onto wound healing also has been shown by applying micron sized bioactive glass particles into open wounds before sewing them: with no increase in inflammatory reaction compared to control wounds, newly formed subcutaneous tissues presented higher breaking strength [79]. Studies have also shown the impact of bioactive glass particles design. For example, nanoporous microparticles induced lower clotting times than dense ones [80], which could be explained by blood capillary adsorption inside the pores of the particles. The particles synthesis process has also been shown to have a huge impact on wound healing: sol-gel-derived particles dispersed in Vaseline and used as an ointment onto full-thickness skin wounds led to a faster healing than melt-derived particles [81]. As for hard tissues regeneration, a larger specific surface area is thus beneficial. However, micron-sized particles presenting a nano-structuration and well-separated 30 nm-sized particles both made using sol-gel chemistry had the same wound healing efficiency, which can be easily understood: as with blood thickening, particles should quickly aggregate, only retaining a global nano-structuration.

Thanks to their antibacterial and hard tissue regeneration properties, bioactive glass nanoparticles are promising materials for applications in dentistry. As mentioned before, bioactive glass dissolution leads to an increase in pH providing good antibacterial properties [82]. Waltimo *et al.* showed the advantage of using bioactive glass nanoparticles for teeth root canal disinfection through a drastic increase in killing efficacy of *E. faecalis* using 30 nm-sized compared to 100 µm-sized particles [83]. Concerning hard tissue regeneration, bioactive glass particles have been studied for dentine remineralization and dentine repair to solve hypersensitivity problems. Again, a particle size in the nanometric range is highly beneficial. Vollenweider *et al.* showed that 30–50 nm-sized particles (170 mg/mL, dispersed in water) induced a faster remineralization of Ethylenediaminetetraacetic acid (EDTA)-demineralized teeth compared with ~100 µm-sized particles [84]. As for the antibacterial effect, the better efficiency of nanometric bioactive glass particles arises from an increase in specific surface area and thus an increase in dissolution rate. The use of nanoparticles has also been shown to be promising for hypersensitivity treatment as nanoparticles are able to penetrate dentine exposed tubules rather than just remaining onto dentine surface [85]. For this study, particles were dispersed in saliva and the slurry was mechanically brushed onto the teeth for 2 min. Then the teeth were rinsed and stored in saliva for 24 h at 36 °C. Using nanoparticles, rods of apatite grew from the exposed tubules, obstructing them, whereas microparticles led to a surface apatite layer onto the teeth. Bioactive glass nanoparticles are thus very promising to provide a longer-term solution to hypersensitivity problems, as apatite rods would be more difficult to dislodge through brushing and normal eating processes.

## 4. Conclusions

With an increasingly better comprehension of sol-gel processes, it is now possible to synthesize bioactive glass nanoparticles with a desired size. Some progress is, however, still necessary in order to control their dispersity in size and to prevent their agglomeration. Tailored porosity can also be obtained while playing onto surfactant nature, concentration and synthesis protocol.

A broad range of bioactive glass nanoparticles-based materials have been presented in this review, focusing on their biomedical advantages compared to bulk or micro-sized-based materials. Most of their advantages lie in their superior specific surface area, leading to higher dissolution rate and thus faster apatite formation and ions/drugs release. Dispersed inside a polymeric scaffold, they have been shown to increase the mechanical properties of such composite, which are now reaching those of natural bones, and to provide biomimetic nano-structuration enhancing cell adhesion.

Although some work still needs to be done in order to design materials perfectly matching biomedical purposes, most of the materials presented have shown good biocompatibility and no cytotoxicity, paving the way for *in vivo* tests.

## Figures and Tables

**Table 1:** Comparison of experimental conditions for different sol-gel syntheses protocols.

Strategy	Reference	Acid	PO_4_^3−^ Precursor	Water:EtOH Vol. Ratio	Si:Ca(:P)	[Tetraethyl Orthosilicate (TEOS)] (mol/L)	Drying	Annealing
1	[14]	/	/	0.11:1	0.43:0.57	0.045	Not detailed	680 °C
	[15]	HNO_3_	/	0.39:1	0.74:0.26	0.14	80 °C 24 h	700 °C 2.5 h
2.i	[16]	citric acid	/	12.7:1	0.70:0.30	0.043	freeze-drying	700 °C
	[15]	citric acid	/	13.2:1	0.74:0.26	0.043	80 °C 24 h	700 °C 2.5 h
	[18]	HNO_3_	(NH_4_)_2_HPO_4_	26.7:1	0.58:0.37:0.05	0.031	25 °C 24 h	650 °C 3 h
	[19]	citric acid	(NH_4_)_2_HPO_4_	27.5:1	0.52:0.38:0.10	0.026	Not detailed	700 °C 3 h
	[17]	citric acid	(NH_4_)_2_HPO_4_	8.03:1	0.39:0.35:0.26	Not detailed	60 °C 8 h	700 °C 6 h
2.ii	[20,21]	HNO_3_	Et_3_PO_4_	0.33:1	0.61:0.36:0.03	1	80 °C 48 h	700 °C 3 h
	[22]	HNO_3_	Et_3_PO_4_	1.2:1	0.55:0.38:0.07	0.96	130 °C 24 h	600 °C 4 h
	[23]	HNO_3_	Et_3_PO_4_	0.33:1	0.57:0.35:0.08	1.1	60 °C 24 h	600 °C 2 h

**Table 2:** Morphology, composition, specific surface area, pore size and arrangement of mesoporous bioactive glasses from the literature, along with the surfactant used for their synthesis.

Reference	Morphology	Size	Composition Si:Ca(:P)	Surfactant	Specific Surface Area (m^2^/g)	Pore Size (nm)	Pore Arrangement
[37]	Microsphere	1 mm	0.80:0.15:0.05	P123 9.2 mM	336	5	Hexagonal
[38]	Monoliths	/	0.95:0.05	P123 9.2 mM	338	5.5	Hexagonal
			0.84:0.16		229	5.2	
			0.73:0.27		147	5.2	
			0.63:0.37		159	4.6	
[32]	Hollow nanoparticles 250 nm	250 nm	0.80:0.15:0.05	Hexadecyl trimethylammonium bromide (CTAB) 6.9 mM	949	2.6	Vertical mesochanels in the shell
[36]	Polydispersed nanoparticles	Mean size of 400 nm	0.82:0.09:0.09	CTAB 28.1 mM	484	1.1 + 3.7	Defective order
				P123 1.8 mM	380	3.9	
				F127 0.8 mM	275	3.5	Hexagonal
[31]	Hollow nanoparticles	294 nm	0.77:0.15:0.08	CTAB 3.3 mM	444	8.8	Not detailed
	Hollow nanoparticles	264 nm		CTAB 4.6 mM	600	5.6	Not detailed
	Dense nanoparticles	187 nm		CTAB 5.9 mM	972	4.6	Not detailed
[30]	Nanoparticles	150 nm	0.77:0.15:0.08	CTAB 1 mM	318	3.7	Worm-like
	Nanorods	150 × 380 nm		CTAB 3 mM	388	3.7	Worm-like
	Nanorods	150 × 550 nm		CTAB 6 mM	455	3.7	Hexagonal
[24]	Nanoparticles	30 nm	0.79:0.17:0.04	CTAB 1.7 mM	1040	2.2	Worm-like
[33]	Nanoparticles	133 nm	0.58:0.35:0.08	CTAB 35.6 mM	684	5.1	Radial mesostructure
	Nanoparticles	234 nm			349	7.8	
	Nanoparticles	254 nm			259	11.2	
	Pineal particles	28 nm			151	9.9	Lamellar mesostructure
	Pineal particles	161 nm			280	10.5	
	Pineal particles	193 nm			192	14.0	
